# *Enterococcus faecium* Regulates Honey Bee Developmental Genes

**DOI:** 10.3390/ijms222212105

**Published:** 2021-11-09

**Authors:** Yating Du, Shiqi Luo, Xin Zhou

**Affiliations:** 1 College of Food Science and Nutritional Engineering, China Agricultural University, Beijing 100083, China; bs20193060574@cau.edu.cn; 2 Department of Entomology, College of Plant Protection, China Agricultural University, Beijing 100193, China

**Keywords:** *Enterococcus faecium*, gut bacteria, honey bee, miRNA, transcriptome

## Abstract

Honey bees provide essential pollination services to the terrestrial ecosystem and produce important agricultural products. As a beneficial lactic acid bacterium, *Enterococcus faecium* is often supplied as a probiotic for honey bees and other animals. However, the underlying mechanisms of its actions and possible safety risks are not well understood. We present the first complete genome sequence of *E. faecium* isolated from the honey bee gut using nanopore sequencing, and investigate the effects and mechanisms of interactions between *E. faecium* and honey bees via transcriptome and miRNA analysis. *E. faecium* colonization increased honey bee gut weight. Transcriptome analysis showed that developmental genes were up-regulated. In accordance, the target genes of the down-regulated miRNAs were enriched in developmental pathways. We describe how *E. faecium* increases honey bee gut weight at the transcriptional and post-transcriptional levels, and add insights about how miRNAs mediate host and bacteria interactions.

## 1. Introduction

Honey bees (*Apis mellifera*) create enormous economic value through products such as honey and wax, but more importantly as pollinators [1]. The use of probiotics as colony additives is a widely accepted approach to improve honey bee health [2,3]. Most probiotics are isolated from honey bee guts or colony-related environments, belonging to the genera *Lactobacillus*, *Bifidobacterium*, *Enterococcus*, and *Bacillus* [2,4]. Strains of *Lactobacillus* reduced pathogenic *Nosema ceranae* spore loads in adult honey bees by up-regulating expression of key immune genes. They decreased the mortality associated with *Paenibacillus larvae* infection, which is responsible for American foulbrood disease (AFB) [5,6]. The EFD strain of *Enterococcus* *faecium,* isolated from pollen granules, reduced *P. larvae*, thereby protecting honey bees from AFB [7]. Feeding honey bees with a probiotic mixture of *Lactobacillus* and *Bifidobacterium* controlled *N. ceranae* infection, and increased honey production [8,9,10]. In addition, *Enterococcus* and *Lactobacillus* isolated from foods were used as probiotics against *P. larvae* by increasing honey bee antimicrobial peptide gene expression after oral administration [11]. *Bacillus* spp. administered to bee colonies increased the number of bees and honey storage, and reduced *Nosema* sp. and parasitic *Varroa* mites [12].

Among the probiotic bacteria used in honey bees, *E. faecium* is a generally commensal organism that is commonly found in animal gastrointestinal tracts, fermented food, dairy products, and in various environments including soil and water [13]. *Enterococcus* is common in honey bee colonies [14,15,16] and was first isolated from *A. mellifera* adults in 1972 [17]. *E. faecium* has been found in the gastrointestinal tract of the giant honey bee, *A. dorsata* [18] and in *A. mellifera* [19], confirming the ability of *Enterococcus* strains to persist in the intestinal tracts of other *Apis* genera. *E. faecium* is resistant to bile salts and the harsh conditions of the gastrointestinal tract. It shows auto-aggregation and adhesion ability, and produces a wide variety of bacteriocins called enterocins [20,21]. Bacteriocins are small, heat-stable, ribosomally synthesized antimicrobial peptides active against other bacteria, while the producing bacterium is immune to them [22]. Bacteriocin production is generally regarded as a desirable feature of probiotics [22]. Enterocin is the broad-spectrum antimicrobial bacteriocin derived from *Enterococcus* spp., which shows high antimicrobial activity towards *L. monocytogenes*, *Staphylococcus aureus* and could be used in hospital settings and food to control undesirable microorganisms [23].

Although *E. faecium* strains isolated from hospitals and others from diseased animals have been considered as opportunistic pathogens with strong antibiotic resistance [24], other strains of *E. faecium* have been widely used as probiotics in animal breeding. *E. faecium* has shown varied probiotic effects in different animals, including improving nutrient metabolism [25] and modulating the gut barrier in broiler chickens [26], reducing infection in swine [27], improving growth in fish [28], and protecting honey bees from AFB [7]. However, the molecular interactions at the transcriptional and post-transcriptional levels between probiotic *E. faecium* and animals remain unclear.

MicroRNAs (miRNAs) are small (20–22 nt long) non-coding RNAs that can efficiently regulate gene expression in a sequence-specific manner, via mRNA cleavage or translational repression. MiRNAs play important roles in gene regulation at both post-transcriptional and translational levels [29]. MiRNAs are important regulatory molecules of host-microbiome crosstalk [30]. The microbiome influences host health via regulating certain host miRNAs [31], and the host miRNAs also regulate the bacterial composition [32].

To explore the potential molecular interactions between *E. faecium* and honey bees, we isolated an *E. faecium* strain H7 from the gut of *A. mellifera*, and fed it to the newly emerged honey bee. The H7 colonization increased host gut weight. The transcriptome and microRNA (miRNA) profiles showed the developmental gene regulation at the transcriptional and post-transcriptional levels. This study provides new insights into how a honey bee commensal bacterium increases gut weight. The new genome adds to our understanding of honey bee gut bacteria diversity.

## 2. Results

### 2.1. Draft Genome Sequence of E. faecium Isolated from Honey Bee Gut

**Figure 1:**
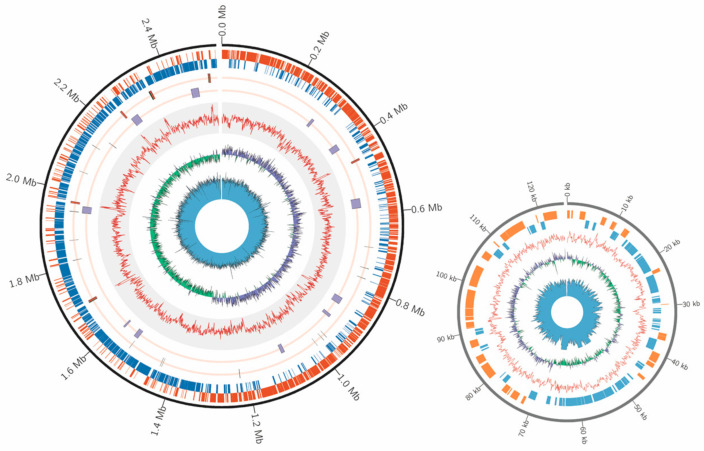
Genome organization of the *E. faecium* H7 strain. Circular overview of the complete chromosome (left panel) and plasmid (right panel) of the *E. faecium* H7 strain. In the chromosome, starting from the outside ring towards the interior: CDS in sense strand (orange) and antisense strand (blue); 6 copies of the ribosomal operon (rRNA, red) and tRNA (black); genome island (purple) and CRISPR (black); GC content (red); GC-skew (+, purple; −, green); sequencing depth (blue). In the plasmid, starting from the outside ring towards the interior: CDS in sense strand (orange) and antisense strand (blue); GC content (red); GC-skew (+, purple; −, green); sequencing depth (blue).

To characterize genomic features of *E. faecium* isolated from the honey bee gut, we sequenced one strain H7 isolated from *A. mellifera*. A total of 142,808 raw reads with a mean read length of 16,983 bp were generated by the Nanopore GridION sequencer, and 8,621,048 raw reads by the Illumina NovaSeq 6000 platform. One complete circular chromosome (2,529,646 bp) and a plasmid (124,878 bp) were assembled (Figure 1), representing the first complete genome sequence for *E. faecium* isolated from *Apis* gut. A total of 2509 CDS, 69 tRNA, and 6 copies of the ribosomal operon were predicted in this genome. The GC contents of chromosome and plasmid were 38.15% and 38.28%, respectively.

**Figure 2:**
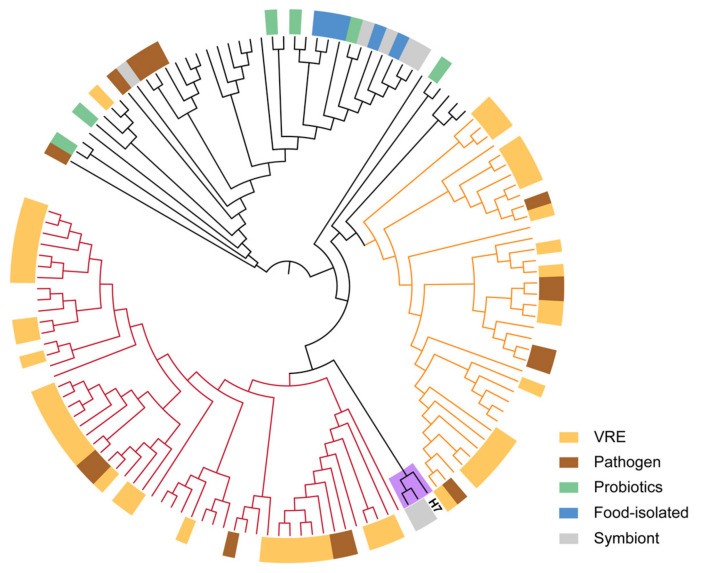
Maximum likelihood tree of *E. faecium* strains. The tree is based on core gene alignment of 136 *E. faecium* strains. H7 was closer to the human symbiont. The groups with dark red or orange branches mainly consist of vancomycin-resistant enterococci (VRE) and pathogenic strains.

**Figure 3:**
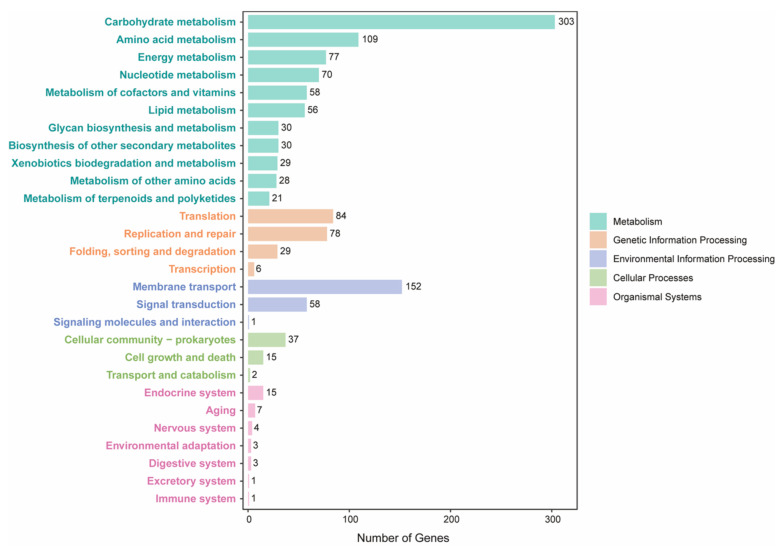
KEGG annotation of *E. faecium* H7 strain. KEGG functions are distributed in different categories. Bars of each functional term indicate the coding gene number for H7.

Phylogenetic analysis of the H7 strain and 37 public genomes from different *Enterococcus* species showed that the H7 strain belonged to *E. faecium* (Figure S1). We also compared the genome sequences of the strain to 135 public *E. faecium* strains (Table S1). Overall, 1316 core genes were present in >99% strains, which were employed in the reconstruction of a maximum likelihood tree (Figure 2). H7 was closer to human commensal strains and was separated clearly from clinical or host-associated pathogenic strains. The genome annotations of H7 showed that 1572 (62.65%) genes were assigned to GO terms, while 1370 (54.6%) genes had KEGG orthologs (Figure 3, Table S2). For the genes in KEGG metabolism categories, genes for carbohydrate metabolism were the most abundant, followed by genes for amino acid metabolism, energy metabolism, and nucleotide metabolism. For genetic information processing categories, there were many genes involved in translation, as well as replication and repair. Moreover, genes related to membrane transport belonging to environmental information processing were also more abundant than other categories (Figure 3).

Enterocins A and B were identified in the chromosome of the H7 strain (Table S3). Enterocins A and B have similar inhibitory spectra and act synergistically against Gram-positive bacteria [33]. Thus, the H7 strain isolated from honey bee guts may provide antimicrobial activities.

On the negative side, pathogenic *E. faecium* strains have spread worldwide as they have evolved multidrug resistance, especially to vancomycin, and can transfer drug-resistant genes across strains [34]. They recruit and maintain a variety of gene clusters coding for the biochemical machinery of vancomycin resistance [35]. These traits, however, were not found in the H7 strain. We did not find any vancomycin resistance genes in the strain, including the two notorious genes *vanA* and *vanB*, which are responsible for most vancomycin-resistant enterococci (VRE) outbreaks in human populations [36]. We found no evidence of pathogenicity in the H7 strain.

### 2.2. E. faecium Increased Gut Weight of Honey Bee and Activated Host Developmental Gene Expression

**Figure 4:**
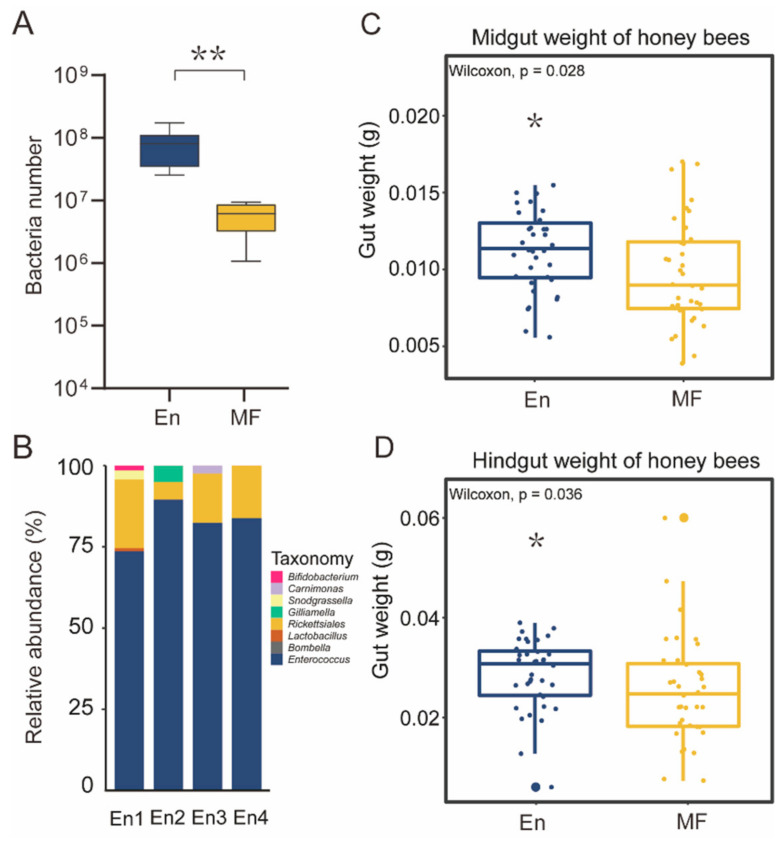
Gut weight in *E. faecium* H7 colonized honey bees. Total bacteria number (En: n = 6; MF: n = 6) (**A**) and gut microbiota composition (n = 4) (**B**) after *E. faecium* H7 colonized for 7 days. The weight of midgut (**C**) and hindgut (**D**) increased in En bees (En: n = 36; MF: n = 36). * *p* < 0.05, ** *p* < 0.01. En: *E. faecium* H7 colonized; MF: microbiota-free.

To investigate how *E. faecium* influences honey bees, we colonized the H7 strain to newly emerged microbiota-free (MF) *A. mellifera* individuals through a feeding experiment. The honey bees colonized with *E. faecium* (En) showed *E. faecium* predominance in the hindgut with up to 10^8^ bacterial cells, significantly higher than MF (Figure 4A,B). H7 colonization did not kill honey bees. Instead, the midgut and hindgut weight of H7 colonized bees were significantly increased compared to MF (Figure 4C,D). A previous study suggested that gut weight gain in the honey bee induced by conventional gut community colonization was correlated to insulin/insulin-like signaling and sucrose sensitivity [37]. However, our behavior test based on the proboscis extension response (PER) showed that H7 colonization did not change the sucrose sensitivity of the honey bee (Figure S2), indicating other mechanisms.

**Figure 5:**
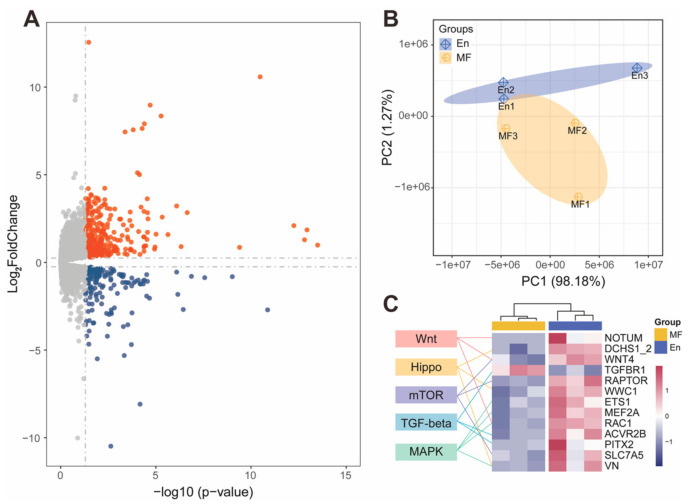
Transcriptome profile of En and MF bees. (**A**) 8925 genes were detected in the RNA sequencing results, with 285 genes significantly up-regulated (red) and 146 down-regulated (blue) in En bee hindgut compared to MF. (**B**) The gene expression profiles of En and MF bee hindgut were clearly separated in the PCA analysis. (**C**) Heatmap of developmental differentially expressed genes (FPKM). Genes involved in Wnt, Hippo, mTOR, TGF-beta, and MAPK developmental signaling pathways were significantly up-regulated in En bees, except the gene TGF-beta receptor type-1 (TGFBR1). En: *E. faecium* H7 colonized; MF: microbiota-free.

The gut microbiota are primarily located in the hindguts of En and MF bees, where they interact with the host. The hindgut transcriptome profiling generated a total of 45.09 Gb raw data with an average of 25 million reads per biological replicate. Pooled replicates showed an average of 94.78% reads mapped to the *A. mellifera* genome assembly HAv3.1 [38] (Table S4). Of the 8925 genes detected in the honey bee hindgut, a total of 431 genes were significantly differentially expressed (DE) after H7 colonization, with 285 genes significantly up-regulated and 146 down-regulated (Figure 5A). Principal-component analysis (PCA) showed a clear separation between the MF and En bees (Figure 5B). The KEGG annotation showed that these DE genes participated in multiple functional pathways (Table S5).

Because gut weights were increased after H7 colonization, we hypothesized that some of the DE genes were related to developmental pathways. Insect gut development relies on complex signal regulation and maintenance. The major developmental pathways regulating *Drosophila* intestinal stem cells include the Wnt, Hippo, MAPK, mTOR, and TGF-beta pathways [39,40]. Interestingly, among the genes in the five developmental pathways, all but one (TGF-beta receptor type-1, TGFBR1) DE genes were up-regulated in En bees (Figure 5C and Table S6). For the Wnt signaling pathway, palmitoleoyl-protein carboxylesterase (NOTUM), protein Wnt-1 (WNT4), and Mig-2-like GTPase Mtl (RAC1) were significantly up-regulated. Up-regulated genes involved in the Hippo signaling pathway included members of the cadherin superfamily protein dachsous (DCHS1_2), ras-interacting protein RIP3-like (VN), and the core gene kibra (WWC1). ETS-like protein pointed (ETS1) and myocyte-specific enhancer factor 2 (MEF2A) from the MAPK pathway were also up-regulated. Regulatory-associated protein of mTOR (RAPTOR) and large neutral amino acids transporter small subunit 2 (SLC7A5) of the mTOR signaling pathway were up-regulated as well (Figure 5C).

### 2.3. MicroRNAs Participated in Host Developmental Pathway Regulation during E. faecium Colonization

For our miRNA sequencing, we obtained a total of 101.95 Mb of raw data with 16,991,859 reads per sample on average. After the removal of reads with low-quality sequences and adaptor trimming, reads between 16–32 nt were retained for analysis (with 15,828,292 reads per sample on average). The length distribution of all retained reads was peaked at 22 nt, covering 30.04% and 21.28% of total clean reads in the En and MF bees, respectively (Figure S3). An average of 89.94% clean reads were mapped to the *A. mellifera* HAv3.1 genome assembly (Table S7). Finally, a total of 127 mature miRNAs were detected in the honey bee hindgut.

**Figure 6:**
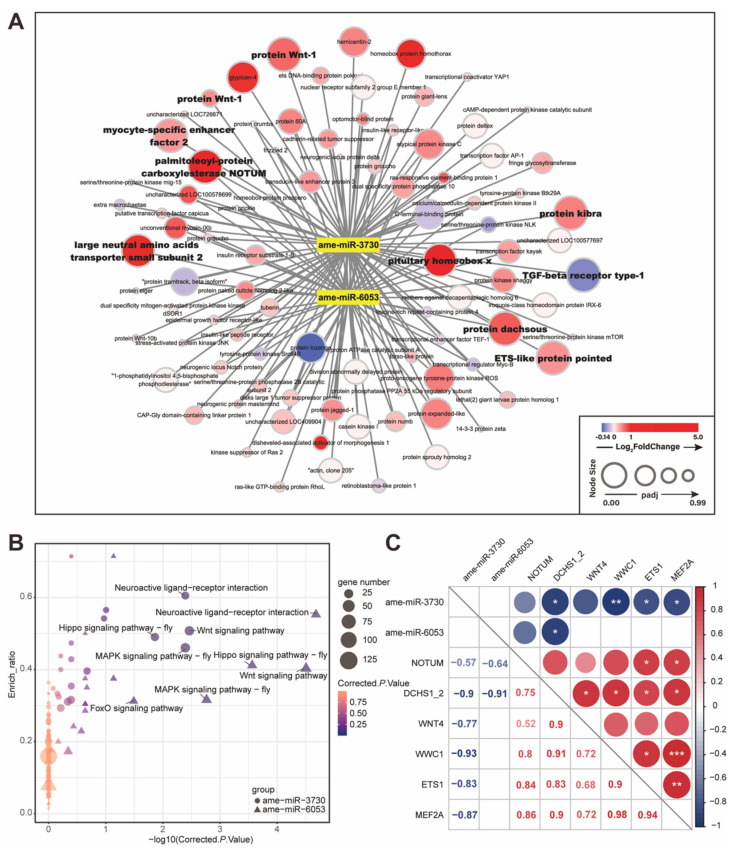
Differentially expressed (DE) miRNAs target developmental genes. (**A**) The network of ame-miR-3730, ame-miR-6053, and 91 predicted target genes in developmental pathways. The node size and color indicate the adjusted *p* value and log_2_fold-change level of target genes in RNA-seq analysis. Target genes belonging to DE gene sets in RNA-Seq are labeled with bold font. (**B**) Target genes of DE miRNAs were enriched in Wnt, Hippo, and MAPK signaling pathways. The area and color of each point are proportional to the gene number and enriched significance (corrected *p*-value). ame-miR-3730: circle; ame-miR-6053: triangle. (**C**) Pearson’s correlation coefficients of miRNA-target gene pairs show the significantly negative relationship between DE miRNAs and developmental target genes. The color of red or blue indicates the positive or negative correlation. * *p* < 0.05, ** *p* < 0.01, *** *p* < 0.001.

Compared to the MF bees, the expressions of ame-miR-3730 and ame-miR-6053 were significantly down-regulated in *E. faecium* colonized bees by more than 2-fold (Figure S4, Table S8). Based on seed region complementarity and minimum free energy in silico, we found that the predicted target mRNAs for ame-miR-3730 and ame-miR-6053 included 91 development-associated genes, 50 of which were shared by the two miRNAs. The expression profile of the development-associated genes in RNA-Seq and their relationship with the two miRNAs were shown in a network (Figure 6A, Table S9). Interestingly, the significantly up-regulated genes associated with development were also predicted as targets of the two miRNAs, such as NOTUM, WNT4, DCHS1_2, WWC1, ETS1, MEF2A, and SLC7A5.

The results of the GO enrichment analysis showed that the predicted target genes of the two miRNAs were involved in similar biological functional terms, and the multicellular organism development term (GO:0007275) was among the top three ranked GO terms (Figure S5, Table S10). The screening against the KEGG database revealed five biological pathways that were significantly enriched. Out of these five pathways, three were development-related pathways including Wnt, Hippo, and MAPK signaling pathways (Figure 6B, Table S10), and were shared by both miRNA targets. Moreover, the FoxO signaling pathway was enriched in ame-miR-6053 target genes, and the pathway associated to neuroactive ligand-receptor interaction was significantly enriched for both miRNA targets.

Combining the results of mRNA and miRNA analyses, we propose that the correlation between miRNA and its direct or indirect target mRNAs is related to host gut development during *E. faecium* H7 colonization. The six genes involved in Wnt, Hippo, and MAPK signaling pathways were significantly up-regulated (Table S6), and contained target sites of ame-miR-3730 or ame-miR-6053. The pair-wise correlation test indicated that four target genes (DCHS1_2, WWC1, ETS1 and MEF2A) showed significantly negative correlations (*p* < 0.05) with ame-miR-3730 or ame-miR-6053 (Figure 6C), while NOTUM expression was also negatively related to the two miRNAs. The results suggest that *E. faecium* H7 colonization causes miRNA down-regulation and up-regulation in target developmental genes, thus activating host gut development and increasing gut weight. The results also imply a potential role of miRNAs in host and bacteria interactions.

## 3. Discussion

Our study has shown the effect of *E. faecium* in increasing host gut weight in honey bees by regulating development-associated genes. However, as *E. faecium* is used in a wide variety of settings, including swine [27], chicken [26], and fish [28] production for human consumption, the safety concern of *E. faecium* cannot be ignored. Certain *E. faecium* strains cause bacteremia, endocarditis, and other infections [41]. In addition, there is a possibility that probiotic bacteria in the gastrointestinal tract might migrate into the host haemocoel when the gut barrier is breached and cause pathogenicity to the host [42]. Thus, it is important to comprehensively assess the safety risks of probiotics before use [43].

Previous studies have revealed the essential roles of gut microbiota in honey bee hosts, such as facilitating pollen digestion [44,45], host development [37], and pathogen resistance [46,47]. Interestingly, the mechanisms of increased gut weight and improved host development varied for different gut bacteria. The conventional mixed gut bacteria of honey bees influence insulin/insulin-like signaling pathway and increase gut weight gain as well as sucrose sensitivity [37]. *Bifidobacterium asteroids* stimulate the accumulation of host-derived prostaglandins and juvenile hormone derivatives known to impact bee development [48]. Our results indicate that *E. faecium* changed the mRNA and miRNA expression of development-associated genes. Similarly, microbiota stimulated gut epithelium renewal and changed gut morphology in *Drosophila melanogaster* by activation of JAK/STAT and epidermal growth factor receptor (EGFR) pathways [49]. In summary, the results indicate the intensive crosstalk between gut microbiota and the host.

Interestingly, two important development regulator proteins WNT4 and VN were significantly up-regulated and involved in multiple signaling pathways. WNT proteins act as directional growth factors that orchestrate patterning, expansion, and differentiation of tissues in the organized formation of body plans, and are central regulators of stem and progenitor cell development and maintenance during embryogenesis and adult homeostasis [50]. The gene VN codes RIP3-like protein belonging to the Ras family, and activated Ras proteins stimulate numerous downstream signaling pathways that regulate a wide range of cellular processes, including proliferation, cytoskeletal function, chemotaxis, and differentiation [51]. These results indicate that *E. faecium* colonization might increase honey bee gut weight via up-regulating developmental signaling pathways.

MiRNAs are involved in gut morphology and development. Mice deficient for all miRNAs in the intestinal epithelia showed a goblet cell decrease in the colon and a dramatic increase in apoptosis in the crypts of the large and small intestine [52]. In *Drosophila* gut, miR-305 regulated the Notch and insulin pathways in the intestinal stem cells, which is required for gut homeostasis [53]. By September 2021, a total of 259 miRNAs of *A. mellifera* were recorded in miRbase [54] (https://www.mirbase.org/, accessed on 9 September 2021) and miRNAs play important roles in honey bee development [55], immunity [56], behavior [57], and caste differentiation [58]. We identified ame-miR-3730 and ame-miR-6053 down-regulation after *E. faecium* H7 colonization in the honey bee hindgut, which target genes enriched in Wnt, Hippo, and MAPK signaling pathways. Our study provided evidence of a new role for miRNAs in the host–microbiota interaction in honey bees.

## 4. Materials and Methods

### 4.1. Bacterial Strain Isolation and Culture

The *E. faecium* H7 strain was isolated from gut homogenates of *A. mellifera* workers collected from apiaries from the suburb of Beijing (40.04° N, 116.69° E) in May 2018. The whole guts of individual worker bees were dissected and homogenized in glycerol (50%, *v/v* and stored at −80 °C. The frozen gut homogenates were plated onto brain heart infusion agar (BHIA, CM1136; Oxoid, Hampshire, UK) supplemented with de-fibrinated sheep blood (5%, *v/v*; Solarbio, Beijing, China). The plates were incubated at 35 °C under a 5% CO_2_-enriched atmosphere for 2–3 days. Single colonies were picked and identified by sequencing the 16S rRNA gene amplified with universal primers 27F (5′-AGAGTTTGATCCTGGCTCAG-3′) and 1492R (5′-GGTTACCTTGTTACGACTT-3′) [59].

### 4.2. Bacterial DNA Extraction and Genome Sequencing

The *E. faecium* H7 strain was cultured in BHIA plates supplemented with de-fibrinated sheep blood and incubated at 35 °C under a 5% CO_2_-enriched atmosphere. The whole genomic DNA of pure cultures of the H7 strain was extracted using the CTAB method [60]. Total DNA from the strain was sequenced with the Illumina NovaSeq 6000 platform (350 bp insert size; 150 bp read length; paired-ended [PE]), and with a Nanopore GridION sequencer.

### 4.3. Bacterial Genome Assembly

For GridION sequencing data, clean data were first assembled using Canu [61] (https://github.com/marbl/canu, accessed on 20 April 2021, version 1.7.11, default parameters). The assembly was then corrected with Illumina sequencing data using Pilon [62] (https://github.com/broadinstitute/pilon/, accessed on 20 April 2021, version 1.22, default parameters). For the looped sequence, we used Circularizer [63] (https://sanger-pathogens.github.io/circlator/, accessed on 20 April 2021, version 1.5.5, parameter: fixstart) to allocate the origin of the sequence to the replication start site of the genome, producing the final genome sequence.

Scaffolds shorter than 1 Mb were mapped to the plasmid database (PLSDB, https://ccb-microbe.cs.uni-saarland.de/plsdb/, accessed on 9 March 2019) using blastn [64] (parameters: -evalue 1e-5 -perc_identity 60 -qcov_hsp_perc 90 -max_target_seqs 100,000 -max_hsps 1). The scaffolds with more than 20% of total length mapped were considered as plasmid sequences.

### 4.4. Structural and Functional Annotations for Bacterial Genomes

For genome structural annotation, protein-coding genes were predicted by Prodigal [65] (http://compbio.ornl.gov/prodigal/, accessed on 20 April 2021, version 2.6.3, parameters: -p None, -g 11), and genes with only complete CDS were retained. tRNA genes were predicted using tRNAscan-SE [66] (http://trna.ucsc.edu/tRNAscan-SE/, accessed on 20 April 2021, version 2.0, parameters: -B -I -m lsu, ssu, tsu) and rRNA genes were predicted using RNAmmer [67] (https://services.healthtech.dtu.dk/service.php?RNAmmer-1.2, accessed on 20 April 2021, version 1.2, parameter: -S bac). For other ncRNAs, we used Infernal [68] (http://eddylab.org/infernal/, accessed on 20 April 2021, version 1.1.2, parameters: --cut_ga --rfam --nohmmonly) to map sequences to the Rfam database (http://rfam.xfam.org/, accessed on 20 April 2021) and the predicted candidate sequences longer than 80% of total length were retained. Genomic islands were annotated using IslandViewer4 [69] (https://www.pathogenomics.sfu.ca/islandviewer/, accessed on 20 April 2021), and CRISPR sequences were searched using CRISPRfinder [70] (https://crispr.i2bc.paris-saclay.fr/Server/, accessed on 20 April 2021). Antibiotic-resistant genes and bacteriocin genes were predicted by CARD tools (https://card.mcmaster.ca/, accessed on 20 April 2021) [71] and BAGEL4 [72] (http://bagel4.molgenrug.nl/, accessed on 20 April 2021), respectively.

For genome functional annotation, protein-coding genes were annotated by Interproscan [73] (https://github.com/ebi-pf-team/interproscan, accessed on 20 April 2021, version 5.30–69.0, parameters: -appl Pfam, TIGRFAM, SMART -iprlookup -goterms -t p -f TSV). The annotation information from TIGRFAMs (http://tigrfams.jcvi.org/cgi-bin/index.cgi, accessed on 20 April 2021), Pfam (http://pfam.xfam.org/, accessed on 20 April 2021), and GO (http://geneontology.org/, accessed on 20 April 2021) databases were retained. We used blastp (parameters: -evalue 1e-05 -outfmt 6 -max_target_seqs 5) to map protein-coding genes to KEGG (https://www.genome.jp/kegg, accessed on 20 April 2021) and refseq (https://www.ncbi.nlm.nih.gov/refseq, accessed on 20 April 2021) databases, respectively. The results with aligned coverage over 30% were kept. Genome visualization was conducted using Circos [74] (http://circos.ca/, accessed on 20 April 2021, version 0.69).

### 4.5. Phylogenetic Analysis

A total of 37 *Enterococcus* genomes were downloaded from NCBI and the unified genome annotations were accomplished by Prokka [75] (https://github.com/tseemann/prokka, accessed on 20 April 2021) with default parameters. We used Roary [76] (https://sanger-pathogens.github.io/Roary/, accessed on 20 April 2021, parameters: -p 20 -r -i 75) to identify the core genes of these *Enterococcus* genomes. A maximum likelihood tree was constructed using RAxML [77] (https://github.com/stamatak/standard-RAxML, accessed on 20 April 2021, parameters: -x 12345 -N 1000 -p 12345 -f a -m GTRGAMMA). A total of 135 complete and assembled *E. faecium* genomes were downloaded from NCBI (Table S2). The same methods for genomic annotation and core gene identification were followed as described above. Core genes were aligned by Roary [76] and a maximum likelihood tree of *E. faecium* strains was built using RAxML [77] with the same parameters.

### 4.6. Generation of Microbiota-Free (MF) and E. faecium-Colonized (En) Bees

The protocol for honey bee colonization described by Powell et al. [78] was followed. In brief, late-stage pupae were removed manually from the brood frames of two different hives collected in Beijing, China, and were placed in sterile plastic bins. The pupae were allowed to emerge in a growth chamber at 35 °C and 95% humidity. Ten to twenty newly emerged individual workers were kept in an axenic cup with a removable base and ventilation holes [79], and were fed with sterilized sucrose syrup (50%, *w*/*v*) and gamma-irradiated (30 kGy) sterile pollen. The En bees were obtained by feeding *E. faecium* suspension (OD_600_ = 1) cultured in BHIA plates, which were mixed with food. Each bee was immobilized at 4 °C, and the midgut and hindgut of MF or En bees were dissected and the weights were measured. The gut samples were stored at −80 °C. Honey bee responsiveness to sucrose was conducted using the proboscis extension response (PER) protocol described in Zheng et al. [37].

### 4.7. Extraction of DNA and RNA from Honey Bee Hindgut

Total DNA from the honey bee hindgut was extracted using the CTAB method [60] and was subject to 16S amplicon sequencing and quantitative PCR. For both En and MF groups, total RNA from three bee hindguts was individually extracted using TRIzol [80] and combined as one biological replicate. Three such biological replicates were subject to RNA and small RNA sequencing.

### 4.8. Quantitative PCR (qPCR) for Determination of Gut Bacterial Loads

Universal 16S rRNA gene primers Uni-F (5′-AGGATTAGATACCCTGGTAGTCC-3′) and Uni-R (5′-YCGTACTCCCCAGGCGG-3′) [48] were used to amplify the 16S rRNA gene for each gut sample on a StepOnePlus instrument (Applied Biosystems, Thermo Fisher Scientific, Waltham, MA USA). To determine the absolute bacterial quantity in the samples, we cloned 16S target sequences into plasmid (pEASY^®^-T1 Simple Cloning Kit; TransGen, Beijing, China). The copy number of the plasmid was calculated, serially diluted, and used as the standard. The thermal cycling conditions were set as follows: denaturation stage at 50 °C for 2 min followed by 95 °C for 2 min, 40 cycles of denaturation at 95 °C for 15 s, annealing/extension at 60 °C for 1 min. Melting curves were generated after each run (95 °C for 15 s, 60 °C for 20 s, and increments of 0.3 °C until reaching 95 °C for 15 s). Each reaction was performed in triplicate in a total volume of 10 μL (0.2 μM forward and reverse primer, 1 μL DNA template, and 1 × ChamQ Universal SYBR qPCR Master Mix, Vazyme, Nanjing, China). Each plate contained a positive control and a negative control with water as the template [48]. The total number of bacteria in the gut was calculated by dividing the number of the 16S rRNA genes by 4, which represents the average copy number of 16S rRNA per genome for most bee gut bacteria [48].

### 4.9. 16S Amplicon Sequence Analysis

The V3-V4 amplicons of the 16S rRNA gene were sequenced on the Illumina HiSeq 2500 platform (PE250). Quality filtering and microbiome composition analysis were performed using QIIME2 [81] following established protocols.

### 4.10. RNA-Seq Analysis

The RNA extracted from the hindgut of honey bees was sequenced using an Illumina Hiseq X Ten platform (PE150). RNA-Seq analysis followed Pertea et al. [82]. Raw reads with low quality (quality < 20 in more than 10% base) or containing > 5 Ns were filtered using fastp [83] (https://github.com/OpenGene/fastp, accessed on 20 April 2021, version 0.20.1, parameters: -q 20 -u 10). Clean reads were mapped to the *A. mellifera* reference genome HAv3.1 (GenBank accession number GCA_003254395.2) using HISAT2 [84] (http://daehwankimlab.github.io/hisat2/, accessed on 20 April 2021, version 2.1.0, parameters: --dta-cufflinks --no-mixed --no-discordant -I 1 -X 1000). Subsequently, a gene count table was obtained using StringTie [85] (https://ccb.jhu.edu/software/stringtie/, accessed on 20 April 2021, version 2.0.6, default parameters) and differentially expressed genes were calculated using DESeq2 [86] (https://bioconductor.org/packages/release/bioc/html/DESeq2.html, accessed on 20 April 2021, R version 3.6.2, DESeq2 version 1.26.0). Genes with adjusted *p* value < 0.05 and |log_2_fold-change| ≥ 0.25 were considered as significantly differentially expressed.

### 4.11. Small RNA-Seq Analysis

Small RNA was sequenced using an Illumina Nova-SE50 platform. Raw reads were filtered with fastp [83] (https://github.com/OpenGene/fastp, accessed on 20 April 2021, version 0.20.1, parameters: --adapter_fasta -q 20 -u 10). The reads with low quality (quality < 20 in more than 10% base) or containing > 5 Ns were discarded, while adapter and poly- A/T/C/G were trimmed. Reads between 16 and 32 nt were aligned to *A. mellifera* reference genome HAv3.1 using Bowtie [87] (http://bowtie-bio.sourceforge.net/index.shtml, accessed on 20 April 2021, version 1.2.3, parameters: -p 10 -v 1). Read counts of each miRNA were generated by HTSeq [88] (https://htseq.readthedocs.io/en/master/, accessed on 20 April 2021, version 0.12.4, default parameters). Differentially expressed miRNAs (with adjusted *p* value < 0.05 and |log_2_fold-change| ≥ 1) were calculated using DESeq2 [86] (https://bioconductor.org/packages/release/bioc/html/DESeq2.html, accessed on 20 April 2021). Targets of miRNAs were predicted by MiRanda [89] (https://bioweb.pasteur.fr/packages/pack@miRanda@3.3a, accessed on 20 April 2021, version 3.3a, parameters: -sc 150 -en -20), RNAhybrid [90] (https://bibiserv.cebitec.uni-bielefeld.de/rnahybrid, accessed on 20 April 2021, version 2.1.2, parameters: -p 1 -b 100 -e -20 -m 100000 -v 3 -u 3 -s 3utr_fly), and Targetscan [91] (http://www.targetscan.org/vert_80/, accessed on 20 April 2021, version 7.2, default parameters). The genes predicted by all three pipelines were considered as the target genes of miRNAs. Functional profiles of miRNA target genes were analyzed and visualized with ClusterProfiler [92] (https://bioconductor.org/packages/release/bioc/html/clusterProfiler.html, accessed on 20 April 2021). The miRNA–mRNA relationship network was visualized with Cytoscape [93] (https://cytoscape.org/, accessed on 20 April 2021). Pearson’s correlation coefficients of miRNA-target gene pairs were calculated in R with the normalized and log_2_-transformed expression values of genes and miRNAs.

## Data Availability

Raw data for RNA-seq, small RNA-seq, 16S amplicon sequencing, and bacteria strain genomes were deposited in the BioProject PRJNA760967 in NCBI.

## Supplementary Materials

The following are available online at https://www.mdpi.com/article/10.3390/ijms222212105/s1.
