# Supplementary material for: Enterococcus faecium Regulates Honey Bee Developmental Genes
Source: Int J Mol Sci. 2021 Nov 9;22(22):12105. doi: 10.3390/ijms222212105 (PMC8621553; doi:10.3390/ijms222212105)
Supplement: Supplementary file 1 [file ijms-22-12105-s001.zip › E.faecium_sup_fig_20210922.pdf]

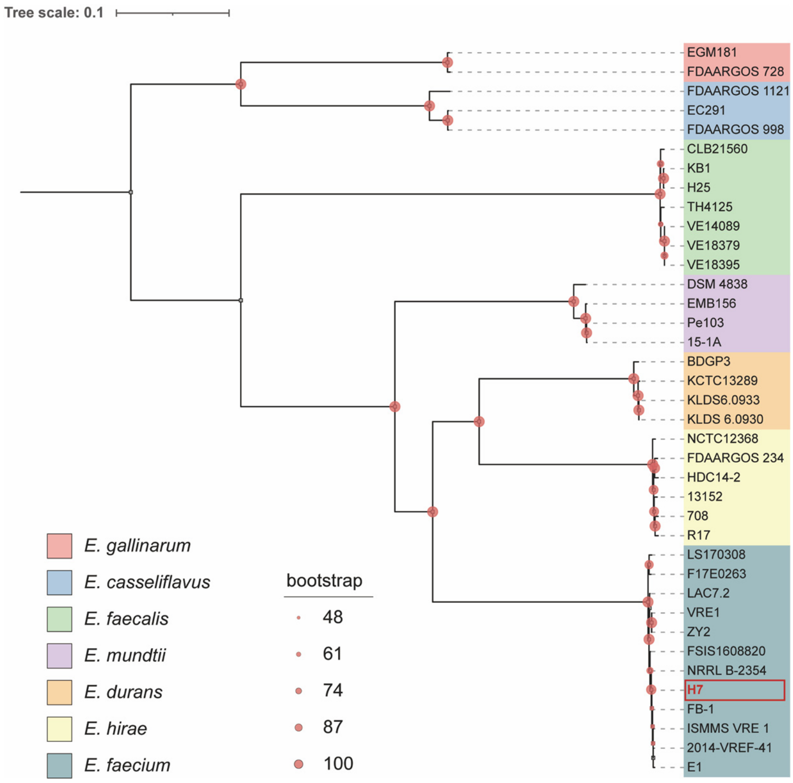

Fig S1. Phylogenetic relationship of H7 strain within *Enterococcus* genus. Core genes of 38 *Enterococcus* genomes were aligned and used for the Maximum Likelihood phylogenetic tree reconstruction. *E. faecium* H7 was marked in red.

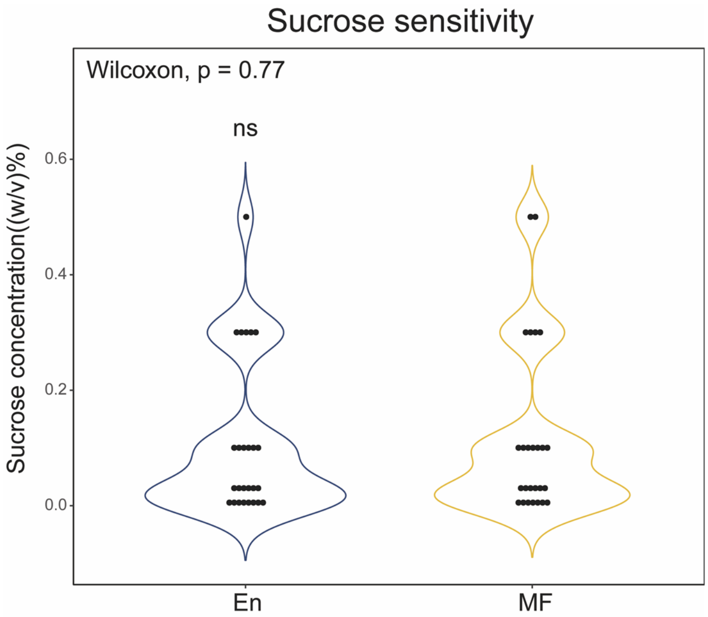

Fig S2. Sucrose sensitivity difference between En and MF bees. En: *E. faecium* H7 colonized (n=26), MF: microbiota-free (n=26). ns, not significant.

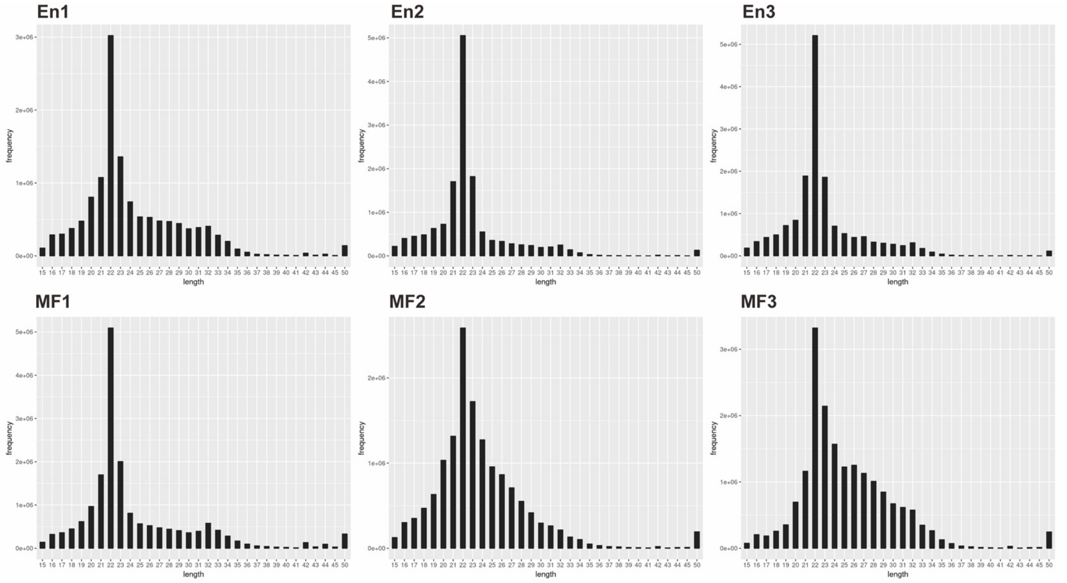

Fig S3. Length distribution of small RNAs in En and MF bees. The peak was at 22nt, which is the typical length of miRNAs. En: *E. faecium* H7 colonized, MF: microbiota-free.

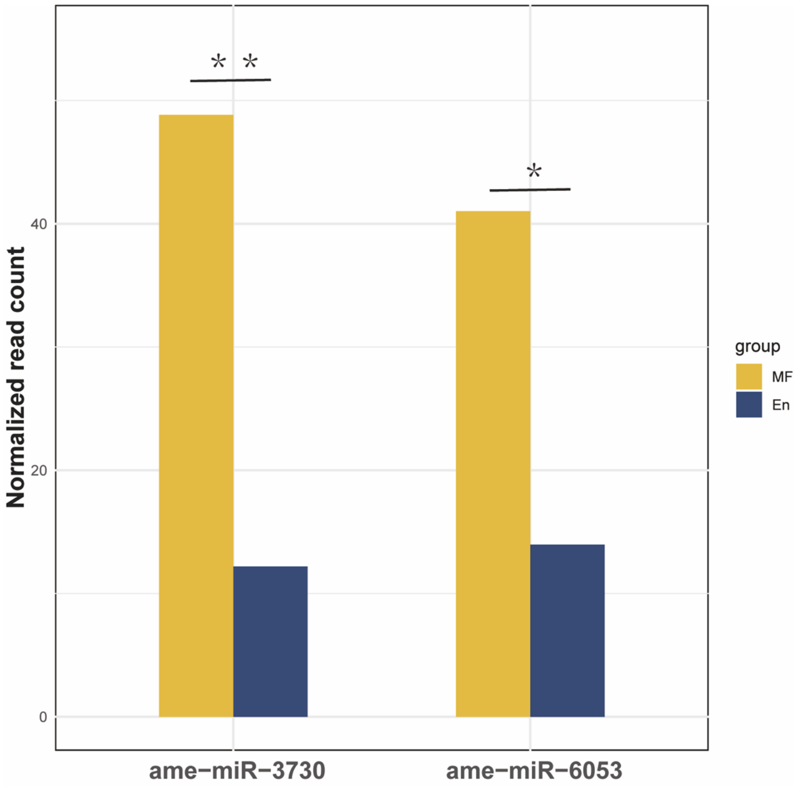

Fig S4. ame-miR-3730 and ame-miR-6053 were significantly down-regulated in *E. faecium* colonized bees. *, $P < 0.05$, **, $P < 0.01$. En: *E. faecium* H7 colonized, MF: microbiota-free.

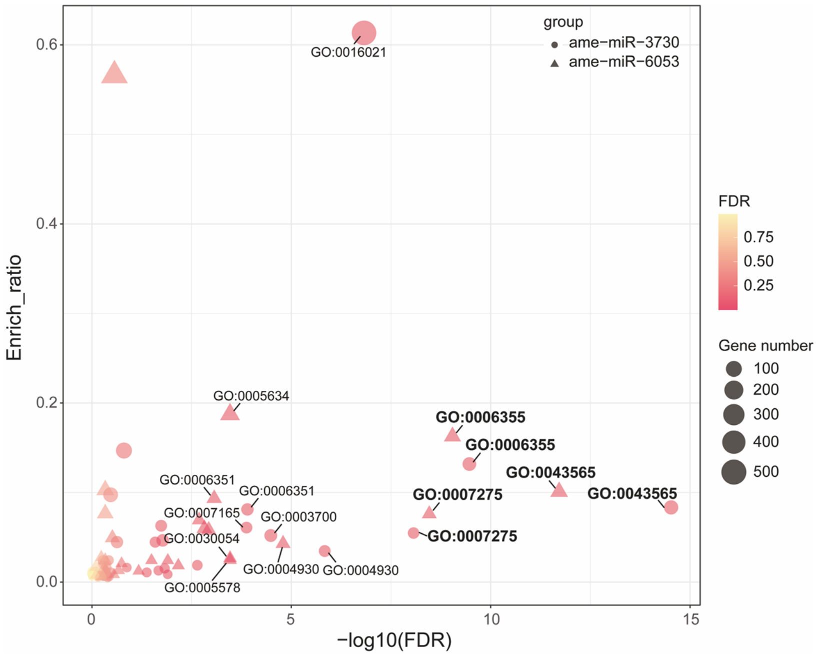

Fig S5. GO enrichment of miRNA target genes. The area and color of each point were proportional to the gene number and enriched significance (FDR). The top three ranked GO terms were sequence-specific DNA binding (GO:0043565), regulation of transcription, DNA-templated (GO:0006355), and multicellular organism development (GO:0007275). ame-miR-3730: circle; ame-miR-6053: triangle.
